# Antigen-driven bystander effect accelerates epicutaneous sensitization with a new protein allergen

**DOI:** 10.1186/1423-0127-16-28

**Published:** 2009-03-06

**Authors:** Li-Fang Wang, Jau-Shiuh Chen, Chih-Jung Hsu, Ching-Yi Liu, Jhang-Sian Yu, Shi-Chuen Miaw

**Affiliations:** 1 Department of Dermatology, National Taiwan University Hospital and National Taiwan University College of Medicine, Taipei, Taiwan; 2 Graduate Institute of Immunology, National Taiwan University Hospital and National Taiwan University College of Medicine, Taipei, Taiwan

## Abstract

Exposure to protein allergen epicutaneously, inducing a Th2-dominant immune response, sensitizes the host to the development of atopic disease. Antigen-driven bystander effect demonstrates that polarized T cells could instruct naïve T cells to differentiate into T cells with similar phenotype. In this study, we aimed to determine the contribution of antigen-driven bystander effect on epicutaneous sensitization with a newly introduced protein allergen. BALB/c mice were immunized intraperitoneally with BSA emulsified in alum, known to induce a Th2 response, three weeks before given BSA and OVA epicutaneously. Lymph node cells from these mice restimulated with OVA secreted higher levels IL-4, IL-5 and IL-13 as compared with cells from mice without BSA immunization. In addition, BALB/c mice immunized subcutaneously with BSA emulsified in complete Freund's adjuvant, known to induce a Th1-predominant response, also induced higher Th1 as well as Th2 cytokine response when restimulated with OVA as compared with mice without immunization. We demonstrated that subcutaneous immunization with BSA in CFA induced Th2 as well as Th1 response. The threshold of epicutaneous sensitization to OVA was also reduced, possibly due to increased expressions of IL-4 and IL-10 in the draining lymph nodes during the early phase of sensitization. In conclusion, antigen-driven bystander effect, whether it is of Th1- or Th2-predominant nature, can accelerate epicutaneous sensitization by a newly introduced protein allergen. These results provide a possible explanation for mono- to poly-sensitization spread commonly observed in atopic children.

## Background

In the past several decades, there has been a progressive increase in the prevalence of atopic disease and an associated increase in the cost of medical management [1]. Atopic diseases are manifested as atopic dermatitis (AD), asthma, and allergic rhinitis. Atopy is associated with the expression of allergen-specific immune response characterized by the production of Th2 cytokines such as IL-4, IL-5, and IL-13; elevated IgE production; and eosinophilia. In contrast, non-atopic individuals display predominant Th1 immune response characterized by the production of IFN-γ. Genetics predisposition and exposure to various environmental allergens, which result in a Th2 immune response, contribute to the pathogenesis of atopic diseases [2].

AD is often the first clinical manifestation of an atopic triad and is the beginning of the "atopic march" [3]. However, the route of sensitization by an allergen that results in AD is still unclear. There is compelling evidence to show that epicutaneous exposure to a protein allergen is one of the important routes to sensitize the host for AD and other atopic diseases [4]. By the presence of cutaneous lymphocyte-associated antigen (CLA)-positive T cells, it is shown in humans, that T cells are primed or reactivated in the skin or its draining lymph nodes (LNs) [5,6]. In AD patients, the increased frequency of prior activation and secretion of type 2 cytokines, as well as higher proliferation response to allergen, are largely confined to a CLA-positive subset [7-9]. Moreover, T cell receptor skewing is only detectable within the CLA-positive subset of T cells from those subjects from whom superantigen-secreting, skin-dwelling bacteria could be identified [10]. The recent demonstration of the expression of CLA by CD8 T cells specific for a skin-tropic, but not non-skin-tropic, virus further supports this concept [11]. We and others have demonstrated in an atopic dermatitis animal model, that epicutaneous exposure of protein antigen induces a predominant Th2 response with high IgE production [12,13]. Furthermore, epicutaneous sensitization with protein antigen induces AD-like skin lesions and development of asthma [14]. The epicutaneously-induced Th2 response requires IL-10 and IL-13 [15,16], while down-regulation of the response is mediated by C3a, cyclooxygenase-2, and skin scratching [17-19].

It has been demonstrated that through an antigen-driven bystander effect, polarized T cells instruct naïve T cells to differentiate into T cells with a similar phenotype. This bystander effect is observed only when the challenge inoculum contains both the original antigen and a newly introduced antigen. This effect was initially shown to be a mechanism of antigen-driven peripheral tolerance after oral administration of antigens, and it can protect rats from developing experimental autoimmune encephalomyelitis [20]. Adoptive co-transfer of two populations of T-cell receptor transgenic T cells of different specificities demonstrated that polarized Th1 or Th2 effector cells can instruct naïve T cells to differentiate into Th1 or Th2 cells, respectively [21]. Subsequently, study of a murine asthma model revealed that an ongoing Th2 response can induce antigen-specific Th2 response to a new antigen, a process termed "collateral priming" [22]. Further exploration of the underlying mechanisms of the antigen-driven bystander effect has demonstrated that conversion of a naïve T cell occurs only when it interacts with the same APC as the memory T cell, and that the orally immunized memory T cells use IL-4 and IL-10 to "educate" APCs, which in turn induce naïve T cells to produce the same cytokines as those produced by the orally immunized memory T cells [23]. In this present study, we demonstrated that antigen-driven bystander effect, despite its Th1- or Th2-predominant nature, accelerates epicutaneous sensitization with a new protein antigen.

## Materials and methods

### Mice and reagents

Six to 10-week-old female BALB/c and TCR-OVA-DO11.10 mice were purchased from the animal center of National Taiwan University Collage of Medicine and kept in a specific pathogen-free environment. All animal experiments were approved by the animal care committee of the Medical College of National Taiwan University. OVA (Grade V), BSA, CFA, and 4-nitrophenyl phosphate (pNPP) were purchased from Sigma-Aldrich (St. Louis, MO). Alum adjuvant was purchased from Pierce (Rockford, IL) and carboxyfluorescein succinimidyl ester (CFSE) was obtained from Invitrogen (Carlsbad, CA). Capture and biotin-conjugated detecting antibodies for IFN-γ, IL-4, IL-5 used in the ELISA were from PharMingen (San Diego, CA). Streptavidine-alkaline phosphatase was purchased from Southern Biotechnology (Birmingham, AL). The murine IL-13 ELISA kit purchased from R&D systems (Minneapolis, MN) was used for determination of the IL-13 content of supernatants.

### Sensitization

Mice were sensitized as previously described [24]. Briefly, 20 μl of OVA (100 mg/ml or serial dilutions) and 20 μl of BSA (100 mg/ml) were placed on the disc of a Finn chamber (Epitest, Tuusula, Finland). This was then applied to an area of shaved skin on the back of a mouse. For each course of sensitization, freshly prepared patches were applied daily from days 1 to 5. For pretreatment, mice received a subcutaneous (s.c.) injection of 100 μg BSA emulsified in CFA over the bilateral side of tail-base or an intraperitoneal (i.p.) injection of 100 μg BSA in alum adjuvant three weeks before sensitization.

### Adoptive transfer

For adoptive transfer of OVA-TCR CD4 T cells, spleen cells from DO.11.10 mice were positively selected for CD4 T cells using CD4 microbeads. Then, 10^7 ^CD4 T cells/ml were incubated with 1 μM CFSE for 10 min at 37°C. Prewarmed FCS-containing PBS was added and washed by cold PBS. Labeled OVA-TCR CD4 T cells (5 × 10^6^) were intravenously injected into BALB/c recipients 24 h before sensitization.

### LN and spleen cell cytokine production and proliferation assay

Ten days after the start of a sensitization course, mice were sacrificed to obtain axillary, subscapular, and inguinal LNs. Pooled LN cells (1 × 10^6^) were cultured in the presence or absence of 100 μg/ml OVA. Supernatants were harvested 48 h later and stored at -80°C. IFN-γ, IL-4, IL-5, and IL-13 content of supernatants was each measured by a standard sandwich ELISA. The limit of detection for IL-4, IL-5, and IL-13 were all 10 pg/ml, whereas that for IFN-γ was 50 pg/ml. For spleen cells, spleens were harvested 3 weeks after sensitization, and pooled spleen cells were stimulated with 100 μg/ml OVA. For the proliferation assay, graded doses of OVA were added. 48 h after the initiation of culture, [^3^H] thymidine was added and the cells were harvested 18 h later.

### Total RNA extraction, cDNA preparation and quantitative real-time PCR

The patched skin and draining lymph node samples were obtained 24, 48, and 72 h after patch co-administration of BSA and OVA. They were frozen with liquid nitrogen and soaked in 1 ml TRIzol Reagent (Invitrogen, Carlsbad, CA, USA). After homogenization, the total RNA was extracted, cDNA was synthesized, and quantitative real-time PCR was performed according to the manufacturer's instructions. Each sample was analyzed in triplicate. The relative cytokine mRNA expression level of each sample was normalized according to its β-actin expression.

## Results

### Antigen-driven bystander effect enhances epicutaneously-induced Th2 immune response to a co-administered new protein allergen

**Figure 1:**
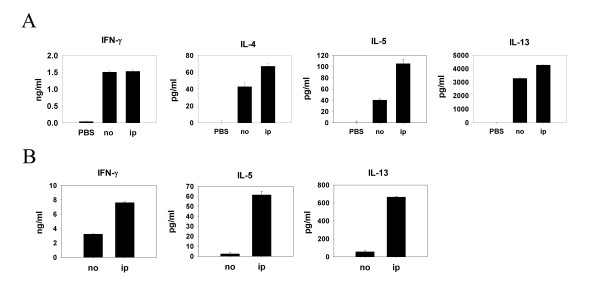
**Antigen-driven bystander effect by Th2 pretreatment enhanced epicutaneously induced Th2 immune response to a new protein antigen**. Groups of mice (four per group) were non-pretreated (PBS, no), or intraperitoneally (ip) pretreated with BSA and alum adjuvant on day -21. They were all epicutaneously sensitized with BSA and OVA (no, ip) or PBS (PBS) on day 1–5. On day 11, draining LNs were obtained (A). On day 22, spleens were taken (B). In vitro reactivation culture with OVA was performed. Cytokine contents of 48 h-supernatants were measured by ELISA. Results were shown as mean ± SEM. Four independent experiments were performed with similar results. One representative results are shown.

**Figure 2:**
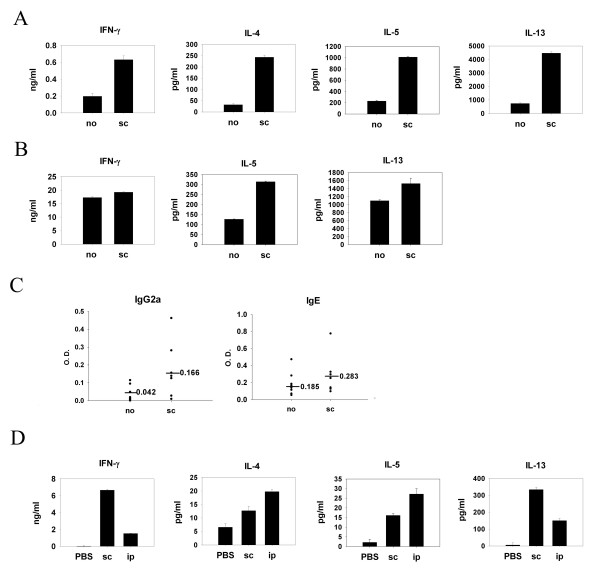
**Antigen-driven bystander effect by Th1 pretreatment also enhanced epicutaneously induced Th2 immune response to a new protein antigen**. (A) (B) (C) Group of mice (Four per group) were non-pretreated (PBS, no) or subcutaneously (sc) pretreated with BSA and CFA adjuvant on day -21. They were all epicutaneously sensitized with BSA and OVA (no, sc) or PBS (PBS) on day 1–5. On day 11, draining LNs were obtained (A). On day 22 spleens were taken (B). In vitro reactivation culture with OVA was performed. Cytokines contents of 48 h-supernatants were measured by ELISA. Results were shown as mean ± SEM. (C) On day 22~26, all mice received another course of OVA patch application. On day 29, blood were collected and serum OVA-specific IgG2a and IgE were determined by ELISA. Three independent experiments were performed with similar results. One representative results are shown. (D) Groups of mice (four per group) were immunized by subcutaneous injection with BSA and CFA adjuvant (sc) or BSA and alum adjuvants (ip) three weeks before their spleens were obtained. In vitro reactivation culture of spleen cells with BSA was performed. Cytokine contents of 48 h-supernatants were measured by ELISA. Results were shown as mean ± SEM. Three independent experiments were performed with similar results. One representative results are shown.

Murine protein-patch model has been established by us and others to study immune responses induced by epicutaneous sensitization with protein antigens [12,14]. It has been shown in previous studies that patch application of OVA solution onto mouse skin induces a predominant Th2 and a weak Th1 immune response. To investigate whether polarized Th2 cells could influence the immune response induced by epicutaneous sensitization with a newly introduced protein antigen, mice were immunized with BSA emulsified in alum adjuvant through the intraperitoneal route and were exposed to both OVA and BSA epicutaneously by patch application three weeks later. Data in Fig. 1A show that LN cells from mice previously immunized with BSA and restimulated with OVA produced significantly higher IL-4, IL-5, and IL-13 in the culture supernatants as compared with cells from mice that did not receive BSA immunization. There was no difference in IFN-γ production between mice receiving immunization and those that did not. Interestingly, when BSA was omitted from the patch, the enhanced Th2 response was no longer observed (data not shown). These results indicate that enhanced Th2 cytokine production was induced by antigen-driven bystander effect. We further tested whether spleen cells also mounted an enhanced Th2-dominant response at a later time point. Splenocytes from mice immunized with BSA and sensitized epicutaneously with OVA and BSA were restimulated with OVA. Fig. 1B shows that splenocyte IL-5 and IL-13 productions were much higher than those from the mice without immunization. However, OVA-specific IL-4 production was not detected in the spleen cell cultures. Next, we tested the antigen-driven bystander effect of polarized Th1 cells. Subcutaneous injection of protein antigen emulsified in CFA adjuvant is a standard way to induce Th1-predominant immune response in experimental murine system. BALB/c mice were immunized subcutaneously with BSA emulsified in CFA or not three weeks before epicutaneous sensitization with OVA plus BSA. LN cells from mice receiving subcutanous immunization of BSA and patch-sensitized with BSA and OVA produced not only higher concentrations of IFN-γ, but also IL-4, IL-5 and IL-13 in the culture supernatants after restimulation with OVA as compared to cells from unimmunized mice (Fig. 2A). In the meantime, the splenocyte IL-5 and IL-13 but not IFN-γ productions were much higher than those from the mice without immunization (Fig. 2B). Mice immunized subcutaneously also had higher levels of serum OVA-specific IgG2a and IgE in the serum (Fig. 2C) than the mice without immunization, although they did not reach statistical significance. As shown in Fig. 2D, subcutaneous immunization with BSA in CFA adjuvant induced both Th1 and Th2 immune responses, indicated by high concentrations of IFN-γ as well as significant amounts of IL-4, IL-5, and IL-13. Taken together, despite its Th1/Th2 predominant nature, the antigen-driven bystander effect enhances the Th2 immune response induced by an epicutaneously introduced new protein antigen.

### Antigen-driven bystander effect decreases the threshold of epicutaneous sensitization with a new protein allergen

**Figure 3:**
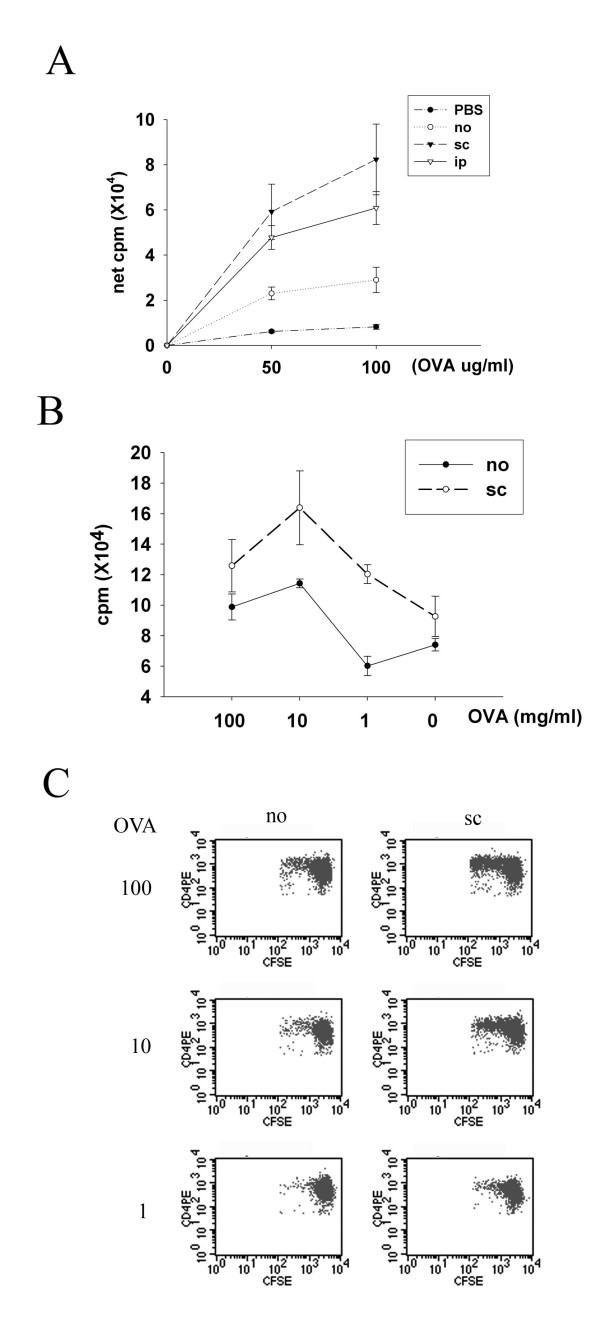
**Influence of antigen-driven bystander effect on the threshold of epicutaneous sensitization with a new protein allergen**. (A) Groups of mice (four per group) were similarly pretreated and immunized as in Fig. 1 (A). On day 11, draining LNs were obtained and OVA-specific proliferation assay was performed. (B) Groups of mice (four per group) were subcutaneously pretreated with BSA (sc) or not (no) on day -21. They received patch co-administration of serial dilutions of OVA (100, 10, 1 mg/ml) and BSA (100 mg/ml) on day 1–5. On day 11, draining LNs were obtained and OVA-specific proliferation assay was performed. (C) Groups of mice (four per group) were non-pretreated (no) or subcutaneously (sc) pretreated on day -21. One day before patch application, all mice received i.v. transfer of CFSE-labeled CD4 T cells prepared from DO11.10 transgenic mice. Patch application with serial dilutions of OVA (100, 10, 1 mg/ml) and BSA (100 mg/ml) were performed on day 1–3. Regional LNs were obtained on day 4 for staining and flow cytometric analysis. All experiments were performed independently for at least three times. One representative results are shown.

The next question was whether the antigen-driven bystander effect could modulate the threshold of epicutaneous sensitization with a new protein allergen. LN cells from mice receiving subcutaneous or intraperitoneal immunization with BSA had higher proliferative response to OVA after patch sensitization with both BSA and OVA as compared with those without immunization (Fig. 3A). Next, serial dilutions of OVA were co-administered with a fixed concentration of BSA to explore whether immunization modulates the threshold of epicutaneous sensitization to OVA in this model system. LN cells from mice subcutaneously immunized with BSA had higher proliferative response to OVA than mice without immunization when OVA was co-administered at concentrations of 100 mg/ml, 10 mg/ml or 1 mg/ml with BSA. Without immunization, OVA co-administered at 100 mg/ml and 10 mg/ml but not at 1 mg/ml with BSA induced cells to respond specifically to OVA stimulation (Fig. 3B). To examine CD4 T cell proliferation in vivo, CD4 T cells from naïve OVA-TCR transgenic mice (DO11.10) were labeled with CFSE and intravenously transferred into BALB/c mice one day before they received patch co-sensitization with OVA and BSA. Fig. 3C shows that transgenic CD4 T cells proliferated in mice subcutaneously immunized with BSA and sensitized epicutaneously with 10 mg/ml and 100 mg/ml OVA plus BSA, and adoptively transferred transgenic CD4 T cells had proliferative response only in unimmunized mice patched with 100 mg/ml OVA (Fig. 3C). Collectively, these data support the notion that the antigen-driven bystander effect decreases the threshold of epicutaneous sensitization with a new protein allergen by at least one order of magnitude.

### The role of cytokines in epicutaneously induced antigen-driven bystander effect

**Figure 4:**
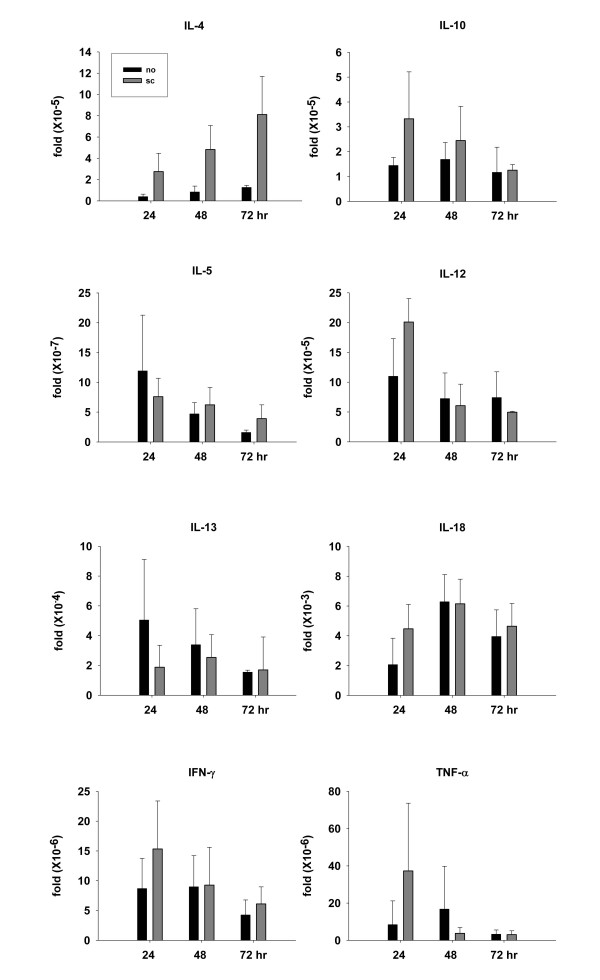
**The role of cytokine in the epicutaneously induced antigen-driven bystander effect**. Groups of mice (three per group) were non-pretreated (no) or pretreated subcutaneously with BSA and CFA adjuvant (sc) three weeks before they received patch sensitization with OVA and BSA on day 1–3. Draining LNs were obtained 24, 48 and 72 hr after start of patch sensitization. Total RNA extraction, cDNA preparation and quantitative real-time PCR were performed individually. The relative cytokine mRNA expression levels of each sample were normalized according to its β-actin expression. Results are shown as mean ± SEM. Three independent experiments were performed with similar results. One representative results are shown.

Among the cytokines that influence the Th1/Th2 balance, IFN-γ, IL-12 and IL-18 are the key cytokines to induce Th1 responses, while IL-4, IL-10 and IL-13 preferentially prime Th2 responses. Figure 4 shows that mice receiving subcutaneous immunization had higher levels of IL-4 and IL-10 expression in their draining LNs as early as 24 h after patch sensitization as compared with mice without immunization. The expressions of other cytokines, including IL-5, IL-12, IL-13, IL-18, IFN-γ and TNF-α, in the draining LNs were similar in mice with or without immunization. It is noteworthy, the expressions of all of these cytokines in the patch-applied skin were not different between mice with or without immunization groups (data not shown). Taken together, these results suggest that immunization-induced IL-4 and IL-10 expression contributes to the epicutaneously induced antigen-driven bystander effect.

## Discussion

The data presented here clearly demonstrate that the antigen-driven bystander effect, despite its Th1- or Th2-predominant nature, accelerates epicutaneous sensitization with a new protein allergen by enhancing the Th2 immune response and by lowering the sensitization threshold. To our knowledge, this is the first report to address the contribution of antigen-driven bystander effect to the epicutaneous sensitization of protein allergens in atopic disease.

The continuation of fetal allergen-specific Th2 responses associated with decreased capacity to produce Th1 cytokine IFN-γ is a defining feature of atopic disease in infancy [25]. After infancy, the major sensitized allergens shift from oral food allergens to aeroallergens [26]. Several observations are consistent with the view that some factors might modulate the susceptibility to allergen sensitization. Firstly, the dose-response relationship between allergen exposure and sensitization differs depending on allergens and locales [27]. Secondly, once sensitized to one allergen, atopic patients are more likely to become sensitized to other allergens [28]. Moreover, there are strong associations of multiple sensitizations both within and between different allergen classes [28]. Skin is continuously exposed to many kinds of allergens and microorganisms in the environment. Microorganisms can gain access to the human body through different routes and sensitize the immune system before contact with the skin. Therefore, it is very likely that the antigen-driven bystander effect affects our skin constantly, although its effect may vary. Thus, the present study provides a possible mechanistic explanation to the clinical observation of the spread of Th2 responses from mono-allergen to multiple allergens in atopic individuals. Another clinical implication of our data is that our results do not conflict with "hygiene hypothesis". The "hygiene hypothesis" predicts that nonspecific suppression of Th2 immune response by a predominant systemic Th1 milieu at the time of sensitization prevents the development of allergy. However, our present study demonstrated specific, but not non-specific, promotion of Th2 immune responses by antigen-driven bystander effect. Collectively, our results support the wisdom of recognizing allergen sensitization early in life and taking precautions to prevent exposure of skins to these allergens.

The mechanism underlying the antigen-driven bystander effect is still obscure. Alpan et al. suggests that orally induced memory T cells utilize IL-4 and IL-10 they produced, but not CD40 ligand, to educate DCs which in turn induce conversion of naïve T cells [23]. However, since co-culturing DCs with naïve T cells plus various concentrations of IL-4 and IL-10 does not simulate the situation in which DC was educated by orally induced memory T cells in their studies, the authors suggested that there must be other factors involved [23]. Our current study provides more information about the underlying mechanism of antigen-driven bystander effect. As we demonstrated in this report, systemic immunization plus skin sensitization to protein antigens induce increased cytokine expression in the draining LNs, but not in the patched skin. This observation supports the notion that the draining LNs are the site where antigen-driven bystander effect takes place [29].

In summary, in this study we demonstrated that antigen-driven bystander effect contributes to accelerated epicutaneous sensitization with a new protein allergen. These results provide a possible explanation for a mono- to poly-sensitization spread commonly observed in atopic children.

## Abbreviations

AD: atopic dermatitis; LN: lymph node; CLA: cutaneous lymphocyte-associated antigen.

## Competing interests

The authors declare that they have no competing interests.

## Authors' contributions

LFW, JSC, CJH and SCM participated in the design of the study and preparation of the manuscript. JSY carried out the measurement of cytokine mRNA expression. CYL performed all other experiments.
